# Elucidation of Complex Atrial Tachycardia Activation in a Patient with Tetralogy of Fallot Using Omnipolar Mapping Technology: A Case Report

**DOI:** 10.19102/icrm.2024.15025

**Published:** 2024-02-15

**Authors:** Jason T. Jacobson, Daniel Frenkel, Daniel Reade, D. Curtis Deno

**Affiliations:** 1 Westchester Medical Center, Valhalla, NY, USA; 2 New York Medical College, Valhalla, NY, USA; 3 Abbott, St. Paul, MN, USA

**Keywords:** Adult congenital heart disease, catheter ablation, intra-atrial re-entrant tachycardia, mapping, omnipolar

## Abstract

In this case report, omnipolar mapping, a unique technology, was used to analyze complex atrial arrhythmias in an adult with congenital heart disease. Our patient had surgically corrected tetralogy of Fallot and presented with highly symptomatic atrial arrhythmias. A successful ablation was performed with standard bipolar mapping techniques. However, due to the complex nature of the substrate and arrhythmias in this patient, bipolar arrhythmia maps were difficult to interpret, and ablation lesions were delivered based on inference and “educated guesses.” An offline re-analysis with omnipolar technology (OT) research software, days after the procedure was performed, revealed details not seen with traditional mapping and explained why the delivered lesions were effective. The findings of this retrospective analysis are provocative, suggesting that OT may increase the accuracy and efficiency of mapping and ablation of complex arrhythmias. Further investigation using commercially released OT in real time is needed.

## Introduction

Atrial arrhythmias (AAs) are not uncommon in patients with adult congenital heart disease (ACHD) and are associated with higher mortality. Ablation is often the first-line therapy for these arrhythmias. Mapping can be challenging due to complex anatomy, extensive atrial scarring, and the propensity for multiple tachycardias with differing mechanisms to be induced in a single patient. High-density mapping catheters have been developed to aid in mapping complex arrhythmias; however, these procedures still require time to generate complete maps, even with automated electrogram (EGM) annotation and mapping algorithms. Standard bipolar (BP) mapping may be limited by difficulty with annotating voltage and local activation time (LAT) due to the orientation of a BP signal in relation to the advancing wavefront. Additionally, multiple scar areas (either due to the hemodynamic effects of the ACHD or surgical incisions) may significantly delay conduction such that activation may belong to a previous cardiac cycle, making isochronal maps confusing. Herein, we report a case of AA mapping in a patient with ACHD presenting just such a problem. In this framework, we also report on a unique mapping tool that allows for real-time wavefront mapping that does not rely on the vagaries of local BP EGM annotation.

## Case presentation

The patient is a 43-year-old man with a history of tetralogy of Fallot who underwent surgical repair as a child, then dual-chamber implantable cardiac defibrillator (ICD) implantation at 37 years of age, and finally mechanical aortic valve replacement at 39 years of age. This patient required repeated emergency department visits due to wide complex tachycardia (some leading to ICD shocks) with rates of 180–200 bpm, with a QRS morphology identical to sinus rhythm (right bundle branch block), suggesting supraventricular (SVT), rather than ventricular, tachycardia. Atrioventricular (AV) node blockade revealed 2:1 atrial tachycardia (AT). The P-wave morphology was consistent with a superior right atrial (RA) origin, essentially ruling out AV node–dependent re-entrant SVT. Clinically, the patient exhibited no signs or symptoms of hemodynamic deterioration from his tetralogy of Fallot. Due to significant symptoms and the increased mortality with AA in patients with ACHD, the patient elected to undergo ablative therapy after a discussion of treatment options and provided informed consent for the procedure.

**Figure 1:**
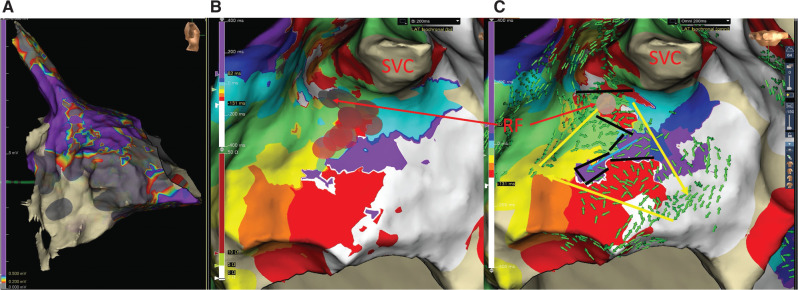
Voltage and activation map AA1. **A:** Right atrial (RA) voltage map. Bipolar (BP) voltage of the RA in the right lateral view. Gray indicates scar < 0.2 mV, and purple indicates normal tissue > 0.5 mV. Note the large scar area on the lateral right atrium from the tricuspid valve to the posterior wall, with patchy extension toward the superior vena cava. **B:** AA1 isochronal BP activation map. White indicates “early,” while purple indicates “late.” Note that there are multiple white zones, and that there is no true dark blue isochrone near the “early-meets-late” zone. The brown disks denote radiofrequency lesions, with the arrow pointing to the successful lesion. **C:** AA1 omnipolar isochronal and omnipolar technology vector (green arrows) map. Note the full representation of all isochrones. The yellow arrows denote the circuit, while the blue arrow indicates conduction into a blind alley. The black bars indicate lines of block. Note the narrow isthmus where radiofrequency ablation (brown dot) changed this tachycardia to AA2. *Abbreviation:* SVC, superior vena cava.

He presented to the electrophysiology laboratory in AT with a cycle length (CL) of 200 ms and 2:1 conduction to the ventricles. Standard BP activation mapping with the EnSite Precision™ cardiac mapping system and Advisor™ HD Grid Mapping Catheter, Sensor Enabled™ (Abbott, Chicago, IL, USA) was limited by a large area of low voltage (<0.2 mV) over the inferolateral right atrium extending posteriorly **(Figure 1A)**. One of the early appearing zones was noted at the superior crista terminalis (CT)/base of the RA appendage **(Figure 1B)** associated with fractionated EGMs. Entrainment attempts would transiently change the tachycardia and so were unhelpful. Irrigated radiofrequency (RF) ablation at this site (TactiCath™ Contact Force Ablation Catheter, Sensor Enabled™; Abbott) slowed the first AA (AA1) to 240 ms, suggesting a change to a second AA (AA2).

**Figure 2:**
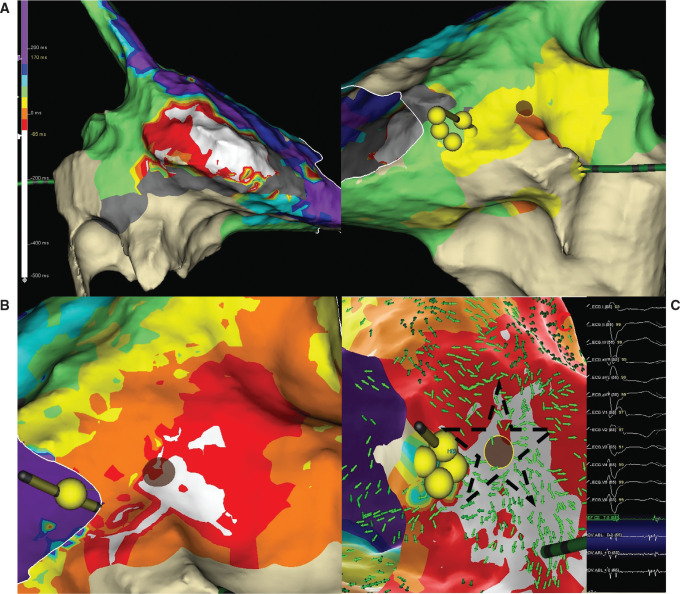
Activation map of AA2. **A:** Bipolar (BP) activation map. Note the broad and variable “early” zone on the lateral right atrial (RA) wall (left panel) and the later area on the septum that has a centripetal activation pattern (right panel). The brown disk is the radiofrequency (RF) lesion that changed this atrial arrhythmia to AA3. The yellow spheres indicate the His bundle. **B:** BP activation map after adjustment of the window. Note that the early activation generally matches the successful RF site. **C:** Omnipolar activation and vector map. Note a similar isochronal pattern to that of the BP map. The starred area highlights the “starburst” pattern of green vector areas all pointing away from the successful RF site. Inset shows the signal at the successful RF site (brown disk).

After mapping the 240-ms AA with the HD Grid, the resulting LAT color pattern provided challenges to interpreting the map **(Figure 2A)**. Considering the possibility of the low-voltage areas leading to interatrial conduction delays affecting the LAT colors, SparkleMap™ (a point-by-point animated propagation map; Abbott) and mapping window adjustments (“early” caliper 50-ms pre–P-wave) were performed to help with interpretation. After the mapping window adjustments were made, the earliest activation was on the RA septum, just posterior to the coronary sinus ostium **(Figure 2B)**. The activation appeared focal; however, a prolonged fractionated potential was recorded here, suggesting micro–re-entry. RF here slowed AA2 to 315 ms, suggesting a change to a third AA (AA3).

Repeat activation and entrainment mapping confirmed this to be a counterclockwise, cavotricuspid isthmus (CTI)-dependent flutter. During RF ablation of the CTI line, AA3 finally terminated to sinus rhythm. With programmed stimulation (double extrastimuli), AA at 350 ms was induced. CTI entrainment confirmed this to be a recurrent typical flutter, and the ablation line was mapped. Repeat RF ablation at a site with small residual EGM terminated the flutter. Bidirectional block was confirmed. No further AAs were inducible. Voltage mapping was repeated in sinus rhythm and confirmed the same area of inferolateral/inferoposterior scar. The patient has had no recurrence of SVT after >6 months.

## Post-processing with omnipolar and LiveView research software

A new mapping technique, EnSite™ Omnipolar Technology (OT) (Abbott), allows for a 360° assessment of local EGMs in order to more accurately determine voltage and activation time. This is achieved utilizing a four-spline, 16-pole grid catheter (HD Grid) and an algorithm comparing EGM signals in multiple orientations within the HD Grid.^1^ Additionally, OT can rapidly determine wavefront directionality across the grid in real time and display this wavefront as vector arrows, which point the operator in the direction of activation.

As this procedure was performed prior to the clinical release of OT, an offline analysis was performed retrospectively on a laptop review station with EnSite Precision™ research software. This enabled comparisons of static BP isochronal and OT vector arrow maps, as well as display activation vectors across the HD Grid in beat-by-beat “real time” (LiveView) during playback of the mapping procedure. All HD Grid unipolar EGMs were obtained with a reference electrode in the inferior vena cava. This analysis was performed days after the procedure and thus was not utilized for patient management.

The BP activation map of AA1 showed a large zone of early activation within the scar region and a more focal early zone at the superior CT **(Figure 1B)**. Targeting this area with RF energy was fortunately effective. The OT map **(Figure 1C)** revealed why this was the case. A re-entrant circuit with a narrow isthmus at the exact site of effective RF ablation is seen, with all isochrones represented. A blind alley within the scar was also revealed by vector arrows colliding along the lines of block. **Figure 1C** identifies the complex circuit as well as the site of successful RF ablation.

**Figure 3:**
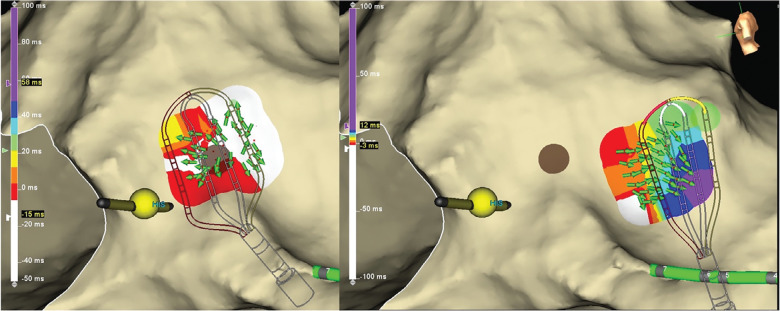
LiveView Omnipolar display of AA2. The left panel displays a “starburst” pattern of green vector arrows when the HD Grid is positioned at the site of origin. The right panel displays the vector arrows pointing away from the sight of origin when the HD Grid is positioned at a remote site.

The standard BP isochronal map of AA2 was difficult to interpret due to activation times in parts of the right atrium exceeding the CL of the AA. This created multiple areas of “early” activation **(Figure 2A)**. This map took 31 min to generate. However, the OT vectors resolved this problem by displaying the wavefront trajectory emanating centrifugally from a focus indicated by a “starburst” of arrows **(Figure 2C)** all pointing away from a site of origin (the successful RF ablation site). Additionally, the LiveView feature displayed this starburst pattern early and often in the mapping session, despite the lack of a complete map **(Figure 3)**. This suggests the potential for a uniquely streamlined procedure.

**Figure 4:**
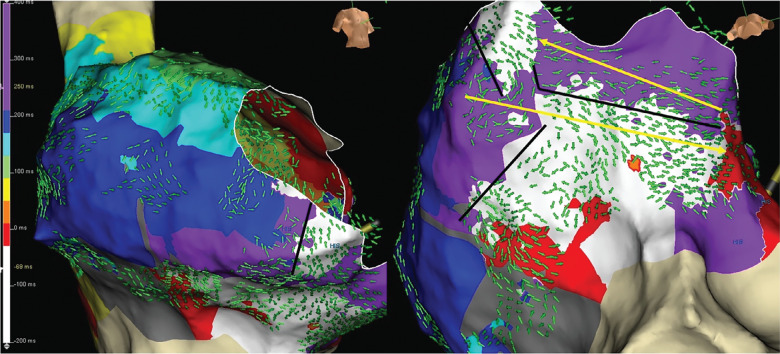
Omnipolar map of counterclockwise cavotricuspid isthmus flutter. RAO (left) and left anterior oblique caudal (right) views of the omnipolar technology activation map and vector arrows. Note the lines of block (black lines) leading to a diagonal wavefront across the cavotricuspid isthmus toward the septum with activation back toward the lateral wall at the tricuspid annulus (yellow arrow).

While the standard isochronal map of the typical flutter showed a simple circuit, the OT map suggested lines of partial block within the isthmus **(Figure 4)**, diagonal to the annulus, running anterolateral to posteroseptal. A possible breakthrough midway to the septum (6 o’clock on the annulus) appeared to allow the wavefront to travel posteriorly before continuing to the septum. The activation of the CTI area proceeded in a complex fashion not fully realized in the linear representation of the BP activation map. This may suggest that a lesion set focused on the gap could have achieved conduction block without requiring a full CTI ablation line.

## Discussion

This case suggests the utility of OT for complex arrhythmia mapping. In addition to the high-density maps obtainable with the HD Grid multipolar catheter, OT allows for greater EGM accuracy by negating the effect of the incident angle between a bipole and wavefront direction. Adding the LiveView vector arrows has the potential to greatly streamline the mapping process. In the retrospective remapping of this case, many new insights were revealed. A full accounting of isochrones and the actual anatomic isthmus became apparent for AA1 with OT activation mapping and OT vectors. Additionally, the complex conduction within scar was demonstrated by the vector arrows; while the isochrones in the areas bordering the isthmus appeared “early” (red and white), the global activation clearly delineated the protected isthmus. For AA2, the HD Grid fortuitously happened over the AT site of origin within the first 6 min. Even without this happenstance, the LiveView vectors literally point away from a focal origin in real time, enabling one to simply trace the arrhythmia. Additionally, this functionality can identify lines of block/colliding wavefronts in real time as well, further resolving the “early-meets-late” color display, which, in some instances, displays very late activation adjacent to a line of block rather than a true circuit. Given the excessive delayed conduction to the lateral wall, activation mapping of AA2 with OT remained complex, while OT vectors showed the way to the focal AT site of origin. Lastly, conduction “leaks” through a partial line of block could be better identified, allowing for a more targeted ablation approach, as suggested by the unusual CTI conduction pattern for AA3.

A strength of this case report is that OT and LiveView were essentially applied in an operator-blinded fashion, as the analysis was done offline after successful ablation. Even though mapping was difficult in this case, success was achieved with traditional techniques and “educated guesses” about ablation targets. While this report does suggest that OT would have streamlined the procedure and reduced the number of RF lesions delivered, this cannot be proven as ablation was not directed by OT. This report is meant to present a provocative, hypothesis-generating analysis of a unique new technology applied to a highly complex arrhythmia substrate, with multiple AAs seen during a single case. A clear limitation of this report is that the interpretation of OT was informed by the outcome of the procedure, potentially overstating the utility OT may have had if utilized during the procedure.

## Conclusion

This case suggests the potential for omnipolar and LiveView mapping technologies to facilitate reductions in the procedure time by rapidly locating the origin of focal tachycardias and reductions in RF lesions by better defining the isthmus of re-entrant tachycardias, respectively. Further investigation using OT in real time will be needed.
